# Removal of the Superior Maxillary Bone

**Published:** 1842-03

**Authors:** 


### ARTICLE IV.

### *Removal of the Superior Maxillary Bone.*

This formidable operation was performed at the Massachusetts General Hospital, by Dr. J. C. Warren, on Saturday, Dec. 4th. The patient, John Farland, had been afflicted some months with the cephalomatous species of carcinoma, commonly known as fungus hæmatodes. The malignant tumor commenced in the left antrum, protruded into the left nostril, breaking down the ossa palati, filling the nasal fossæ, and pressing upwards the inferior orbitar plate. The patient being aware of the nature of the operation, and the chance that it offered for his restoration, submitted to the extirpation of the whole of the disease, with the entire superior maxillary bone, which was effected in the following manner.

The patient being seated in a chair, his head supported, and compression made upon the carotid arteries, an incision was made from the outer angle of the eye to the angle of the mouth, and the flap dissected towards the alæ of the nose, and continued higher up over the cartilages and between the orbit and the eye. The outer flap was dissected in the opposite direction, until the masseter muscle was uncovered, and cut away from the malar bone, about one half of its extent. The orbit was then perforated, and the malar bone cut through. The nasal process of the superior maxillary, near its junction with the sphenoid and ethmoid bones, was next separated; a tooth was removed, breaking down the anterior wall of the alveolus, and the back part was cut through with the forceps. A strong-pointed knife was introduced into the patient's mouth and the soft palate skilfully separated from the palate bones, and the os maxillare detached from the pterygoid process of the sphenoid bone. After the division of the supra maxillary nerve and artery, which was accomplished by cutting from behind forwards, the remaining attachments being slight, the entire mass was removed with but little difficulty or delay. The internal maxillary artery was secured, with a branch of the facial; and the patient, considering the length of the operation, which occupied nearly an hour, suffered less from loss of blood than was anticipated, and is now doing well.

Notwithstanding the frightful cavity made by the removal of this disease, the deformity will be but trifling, should no untoward event occur during the process of uniting the divided parts.

[*Bos. Med. Sur. Journal.*

